# Chicago Medical Society

**Published:** 1868-12

**Authors:** 


### CHICAGO MEDICAL SOCIETY.

FRIDAY EVENING, NOV. 6, 1868.

The meeting was called to order, Dr. Marguerat, President, in the chair.

Dr. McDonald, Secretary, being absent, Dr. Wanzer was chosen *pro tem.*

The name of Dr. Foster, who was recommended for membership at last meeting, had not been acted upon by the Committee, and was deferred.

Dr. E. L. Holmes presented a crystalline lens from the eye of a young man aged 22 years, which dropped into the anterior chamber of the eye while the patient was stooping over to pick up something. The same accident happened to the other eye some two years ago, when the physician in attendance attempted to replace the lens to its natural position by pressure, which resulted in the evacuation of the humors of the eye and collapse. For the past year the patient had suffered considerable pain in the good eye, up to the time of the accident which occurred about one month ago, Dr. Holmes removing the lens on the following day. The patient was always near-sighted, and while the lens occupied the anterior chamber he could still see to read fine diamond print at a short distance. The wound healed one week after the operation, and, to the great delight of the patient, good sight was restored. The Doctor remarked that one week ago there appeared a small hernia near the iris, which he punctured. The eye is still at rest, but doing well.

Dr. H. remarked that the dislocation of the lens was of rare occurrence, and thinks it is due in the present case to congenital malformation, as the patient is a singular-looking fellow, being loose-jointed, especially the hands and wrists, presenting the appearance of one who has had the rickets.

Dr. Holmes also presented a *choroidal tumor*, carcinomatous in character, on the internal portion of the right eye.

Dr. J. P. Ross exhibited a cirrhosed liver taken from a woman who died in the County Hospital, of typhoid fever. This liver was compared to a healthy one; the latter weighing 3 lbs., 12 oz., while the diseased one weighed only 1 lb., 12 oz. The patient was of intemperate habits.

Dr. Ross also presented the lungs of a young man of about 18 years, who died in hospital, of phthisis pulmonalis. Six weeks previous to his entering the hospital he said that he was perfectly well. He died two weeks after entry. The right lung showed quite a cavity in the upper lobe, which was the seat of cavernous respiration during life; the remainder of the lung, as well as the left lung, being thickly studded with tubercle throughout its entire substance.

On admission to hospital, the patient was suffering from cough, night-sweats, hectic, and constant diarrhœa, which continued until death. All other organs seemed in a healthy condition, with the exception of the mesenteric glands, which were slightly enlarged.

The Society then proceeded to the discussion of the subject chosen at the last meeting, *viz.:* "The Therapeutical Value of Carbolic Acid."

The discussion was opened by Dr. Bogue, who gave a brief history of its discovery, etc. Stated that its therapeutical value was lately brought to notice by Dr. Lester, of Edinburgh, who had applied it quite extensively to severe wounds, and in many cases of compound fractures, virtually converting them into simple ones by diminishing, or rather preventing, suppuration. Dr. B. remarked that he had not had any experience in its use in compound fractures.

Stated that the general modes of application were in solution, and in the form of a paste mixed up with whiting. The solution being from 3 to 6 parts of the acid to 20 of water, or linseed or olive oil; which solution, in case of amputation, is to be smeared over the entire flaps before closing; after which, the paste should be applied so as to entirely exclude the air from the wound. Tinfoil has been used for this purpose.

Cases have been reported where no suppuration has occurred under the treatment recommended. Remarked that it had been used in catarrh and ozena, when the discharges were offensive; also as a local application to the mouth and throat, in the strength of from 1 to 3 grs. to the ounce of water or glycerine. Had prescribed it in one case of diarrhœa, characterized by offensive discharges, in $\frac{1}{16}$-gr. doses internally, but without benefit. Stated that the remedy in foul ulcers, etc., was highly spoken of, but that his experience with it had been rather unfavorable; that he had used it in one case of gangrene, and considered it far inferior as an antiseptic to permanganate of potassa, although in this case it might have been owing to the solution being too weak. In cases where there are sinuses or suppurating cavities, the use of 1 part of the crystals to 40 parts of water have been attended with marked benefit.

Dr. B. thinks that it is most applicable and beneficial in lacerated wounds and amputations. Has used it in gonorrhœa and gleet, in solutions of different strength (1 part c. acid to 6 of oil, and 1 part c. acid to 30 of oil), with poor effect. Also used it 1 part acid to 2 of glycerine, on chancres, with very good effect.

Dr. Fisher remarked that he had not had much experience in its use, but that he had used it in one case of compound fracture of the tibia, in the form of paste made with chalk, where there was but little suppuration, and good result; also with benefit in ulcers.

Dr. Ross remarked that he supposed it was purely a surgical remedy, but that he was tempted to use it in one case of chronic dysentery, where the patient was very low.

He gave in this case—

℞. Cupri Sulphas, ---------------------- gr. $\frac{1}{4}$.

Pulv. Opii, -------------------------- gr. j.

alternated with the turpentine emulsion, with injections of a weak solution of carbolic acid, with ʒss. tincture opium. The patient always inquired after the injections, and in two weeks had entirely recovered.

Dr. Quails remarked that he had used it in a case of phlegmonous erysipelas, suppuration stopping on the second day.

Also ten cases of gonorrhœa, 6 acute, 4 chronic, with injections, carbolic acid 1 part, glycerine 12 parts, without very good effect.

Dr. Gray reported case of diarrhœa which was treated successfully by injections, 2 gr. acid to 1℥ water, with gtt. xx. tinct. opii; also, in eczema, with good effect, applied externally.

Dr. Bridge stated that he had used it in one case of ozena, but that the discharge was more copious, although not of an offensive character.

The Society next proceeded to miscellaneous business.

Subject for discussion was then chosen.

Dr. Davis recommended that the subject for discussion two weeks hence be the following, which was duly carried, *viz.:*—"How far do Continued Fevers prevail in this city at the present time, and what are their characteristics?"

The object being to elicit the observations of all members of the Society.

On motion, Society adjourned.

Members present—Drs. Marguerat, Bogue, Davis, Holmes, Loverin, Wanzer, Quails, Avery, Blake, Fisher, Paoli, Ross, Bridge, Gray.

---

FRIDAY, NOV. 13, 1868.

The Society was called to order, President Marguerat in the chair.

The Secretary then read the proceedings of last meeting, which were duly approved and ordered to be placed on file.

Dr. Lyman reported a case which occurred in his practice last summer, where the patient, while getting into a carriage, made a misstep, and was suddenly seized with a severe pain in the vicinity of the trochanter. She was taken home, put to bed, and an anodyne administered. In a day or two the pain was reflected to the anterior portion of the thigh, when the Doctor applied a thimble packed half full of cotton, which was saturated with aqua ammonia. This remedy produced a blister within four or five minutes. The cuticle was then removed, and $\frac{1}{4}$ gr. of morphia was applied to the denuded surface, and the part covered with oiled silk.

This application entirely removed the pain, and the patient was kept in a comfortable condition by resorting to the application twice a day. Small doses of quinine were administered daily, and in a week the patient was able to attend to business. Dr. L. is of the opinion that this method of administering morphia is preferable to hypodermic injection.

Dr. Bogue presented a patient with elephantiasis and chronic eczema, involving one leg from above the knee to the extremity of the toes. The leg was very hard with varicose veins, which were very tortuous. This disease was the result of injury some two years ago. The treatment consisted in an application of iodine, gr. x., glycerine ℥j., to the eczema, and injections of persulphate of iron into varicose veins. Under this treatment the patient has improved considerably.

Dr. Paoli remarked that the case did not look like true elephantiasis to him, as he had seen two cases in which the skin was very much thicker, and darker-colored.

Dr. Lyman remarked that he presumed the color and skin had changed much under treatment; also spoke of having seen two cases of elephantiasis.

Dr. Bogue stated, when the patient presented himself, the skin was much darker-colored, and corresponded to "Wilson's" diagnosis of elephantiasis.

Dr. Wanzer reported two cases:—

Case 1. *Laceration of the Perineum.*—Was called to case where a woman had been in labor twenty hours, with her first child. Found child's head impacted in inferior strait, and labor not progressing. After waiting four hours, introduced forceps. The blades of the forceps were applied in the occipito-frontal diameter of the child's head, and by gentle traction the child was delivered, rupturing the perineum, so as to form a complete opening into the rectum. Immediately gave an anodyne, placed parts in apposition, and kept them so by the application of a couple of sutures. Kept the bowels constipated by use of ten drops tinct. opium, three times a day. Child and mother are both doing well.

Case 2. *Post Partum Hemorrhage.*—Was called in a case of a large, plethoric woman, who had been delivered of a child one hour previously, and who had suffered so severely from hemorrhage as to render her nearly pulseless. At once lowered the head of the bed, so as to produce gravitation of the blood towards the head, and administered fl. ext. ergot ℥ij. in the course of the hour, when all hemorrhage ceased.

Dr. Paoli remarked, that in case of the application of the forceps, it was necessary to support the perineum at time of making traction, and that he should employ an assistant under such circumstances.

Dr. Hatch stated that he did not believe in supporting the perineum where the forceps were used, believing the elevation of the handles all that is necessary, and all that can be done to avoid laceration of the perineum.

Dr. Paoli thinks ergot is not an effectual remedy in producing contraction of the uterus, and would not depend upon it in such serious cases as that reported by Dr. Wanzer; believing there were more reliable remedies in the materia medica.

Dr. Hatch thinks that ergot is one of the most useful articles in case of post partum hemorrhage, it acting as a styptic under such circumstances. In large doses believes it a decided narcotic, diminishing the heart's action. Says he has never failed to produce contraction of the uterus by grasping the fundus with the hand and the administration of ergot, although he has practised medicine forty years. The preparation he generally employed was the fluid extract.

Dr. Paoli says that there is no remedy which will produce contraction in the uterus in case of hemorrhage as efficiently as gallic acid, given in 10-gr. doses. Cited a case of craniotomy, followed by hemorrhage, in which he used a solution of tannin as an injection. Believed that had he depended upon ergot, the hemorrhage would have proved fatal.

Dr. Wickersham stated that gallic acid was not prompt enough in its action, and thinks the physician who neglects the use of ergot in cases of post partum hemorrhage, neglects his very best remedy. Cited a case of hemorrhage where he employed the fluid extract without success, when he resorted to the powder with benefit.

Dr. Merriman remarked that he thought Dr. Wanzer pursued the right course in the administration of ergot, but asked the object of the sutures in the perineum?

Dr. Wanzer said that he thought the ergot had but little to do with stopping the hemorrhage, but believed it due to the lowering of her head and the application of cold water to the hypogastric region.

Dr. Merriman reported the case of a large, plethoric woman, weighing 250 lbs., who, while in labor, suddenly had the pain reflected to her head, the contractions of the uterus being entirely suspended. Waited twenty minutes, and then proceeded to give ergot. While preparing ergot, happened to turn around, and found her jaw set and in convulsions. At once anæsthetized the patient; after which he gave the ergot, and soon delivered her of a child weighing 13¾ lbs. History of patient showed she had convulsions at a previous labor.

Dr. Wickersham thinks that the forceps might have been used, as the traction would have produced contractions of the uterus.

Dr. Loverin said he did not favor the administration of anæsthethics during labor.

Dr. Hatch remarked that he never had anæsthetized a patient when using the forceps, as it was better to have the sensations of the patient to aid you in applying the instruments, thereby avoiding inclusion of any of the soft parts of the mother between the fœtus' head and the forceps.

The case reported by Dr. Merriman was discussed by Drs. Loverin, Paoli, and Wanzer.

Society then proceeded to miscellaneous business, and Dr. A. H. Foster was unanimously elected a member of the Society.

Adjourned.

